# Triglyceride-Glucose Index and Systemic Inflammatory Markers in Polyendocrine Metabolic Ovarian Syndrome (Polycystic Ovary Syndrome): A Cross-Sectional Study

**DOI:** 10.3390/metabo16100771

**Published:** 2026-10-12

**Authors:** Meryem Kececi Oguzhanoglu, Kursat Oguzhanoglu

**Affiliations:** Department of Obstetrics and Gynecology, Haseki Training and Research Hospital, University of Health Sciences, Istanbul 34265, Türkiye; kursatoguzhanoglu@gmail.com

**Keywords:** triglyceride-glucose index, polyendocrine metabolic ovarian syndrome, polycystic ovary syndrome, insulin resistance, systemic immune–inflammation index, C-reactive protein

## Abstract

**Highlights:**

**What are the main findings?**

- In women with polyendocrine metabolic ovarian syndrome (PMOS), the triglyceride-glucose (TyG) index was independently associated with hemogram-derived inflammatory indices after adjustment for body mass index (BMI) and age, whereas C-reactive protein (CRP) was associated only with BMI. Both the triglyceride and glucose components contributed, and fasting insulin was only weakly related to these indices.
- The TyG-inflammation association was consistent across BMI strata, with no evidence of effect modification by BMI in formal interaction tests.

**What are the implications of the main findings?**

- TyG and hemogram-derived indices, obtained from routine and inexpensive laboratory tests, capture a metabolic-inflammatory signal in PMOS that, in this cohort, is independent of adiposity and is also present in normal-weight women.
- CRP alone may be insufficient to represent the metabolic–inflammatory axis in PMOS; whether TyG-associated inflammation carries prognostic value requires prospective evaluation against clinical outcomes.

**Abstract:**

Background/Objectives: Low-grade chronic inflammation is recognized as part of the systemic profile of polyendocrine metabolic ovarian syndrome (PMOS, formerly polycystic ovary syndrome, PCOS), although whether this inflammatory signal tracks insulin resistance, adiposity, or both remains unclear. This study evaluated whether the triglyceride-glucose (TyG) index, a surrogate of insulin resistance, is associated with subclinical inflammation independently of body mass index (BMI) in women with PMOS. Methods: In this retrospective cross-sectional study of 150 women with PMOS, the TyG index was calculated from fasting triglycerides and glucose. C-reactive protein (CRP; standard assay) and the systemic immune–inflammation index (SII) were pre-specified primary markers; the neutrophil-, platelet-, and monocyte-to-lymphocyte ratios (NLR, PLR, and MLR) and the systemic inflammation response index (SIRI) were secondary. Associations were assessed using Spearman correlation and multivariable linear regression adjusted for age and BMI, with component, extended-adjustment, tertile, and BMI-interaction analyses. Results: The TyG index correlated with the homeostatic model assessment of insulin resistance (HOMA-IR) (rho = 0.45, *p* < 0.001) but not with BMI (rho = 0.03, 95% confidence interval [CI] −0.13 to 0.19, *p* = 0.71). After adjustment, each standard deviation (SD) increment in TyG was associated with a 21.7% higher SII (95% CI 17.0 to 26.6%; *p* < 0.001) and with a higher NLR, PLR, MLR, and SIRI (all corrected *p* ≤ 0.023), with no TyG × BMI interaction. Both triglyceride and glucose components contributed, fasting insulin was only weakly associated, and results were unchanged after further adjustment for hormonal and glycemic covariates. TyG was not associated with CRP (*p* = 0.53), which was associated only with BMI. Conclusions: In PMOS, the TyG index was associated with hemogram-derived inflammatory indices independently of BMI, a signal attributable to its triglyceride and glucose components rather than to fasting insulin, whereas standard-assay CRP tracked adiposity. These cross-sectional findings are hypothesis-generating.

## 1. Introduction

Polyendocrine metabolic ovarian syndrome (PMOS) is the updated designation, adopted through international consensus, for what was formerly termed polycystic ovary syndrome (PCOS); it remains the most prevalent endocrinopathy in reproductive-age women, with an estimated prevalence of 8% to 13% [1]. The name change reflects a shift toward viewing the condition as a systemic endocrine–metabolic disorder, while the diagnostic core, the Rotterdam criteria (at least two of oligo-anovulation, clinical/biochemical hyperandrogenism, and polycystic ovarian morphology), remains unchanged [1,2]. Beyond its reproductive manifestations, PMOS is characterized by insulin resistance (IR) largely independent of body weight, alongside increased long-term risk of type 2 diabetes, dyslipidemia, and cardiovascular disease [3].

A second systemic feature of PMOS is low-grade inflammation, intertwined with IR and hyperandrogenism [4,5]. Circulating C-reactive protein (CRP) tends to be higher in PMOS than in controls [6], but whether this reflects the syndrome itself or the excess adiposity that often coexists with it remains debated, since obesity alone is a strong, independent driver of adipose inflammation and CRP synthesis [7]. The triglyceride-glucose (TyG) index, derived from fasting triglycerides and glucose, has gained acceptance as an inexpensive, insulin-independent surrogate for IR, correlating reasonably well with the homeostatic model assessment of insulin resistance (HOMA-IR) and independently predicting cardiovascular risk at the population level [8]; however, this correlation is not uniform across cohorts [9,10,11], and reported discriminative accuracy for IR in PMOS has been modest (area under the curve [AUC] 0.69–0.78) [9,11]. The TyG index has also been linked to inflammatory and adipokine disturbances in metabolic syndrome more broadly [12,13].

Hemogram-derived composite indices, including the systemic immune–inflammation index (SII), neutrophil-to-lymphocyte ratio (NLR), platelet-to-lymphocyte ratio (PLR), monocyte-to-lymphocyte ratio (MLR), and systemic inflammation response index (SIRI), have similarly emerged as inexpensive, readily available measures of systemic inflammatory activity in PMOS, with case-control data reporting elevated values relative to controls [14] and within-PMOS studies linking these indices to clinical phenotype and reproductive outcomes [15,16]. What remains less well characterized is whether the TyG index tracks these hematologic markers independently of body mass index (BMI), rather than the association simply reflecting shared adiposity. Much of the available evidence rests on case-control comparisons between women with PMOS and controls, rather than on within-cohort analyses that examine CRP and hemogram-derived indices side by side with BMI stratification. This matters clinically because a marker such as CRP that rises with both IR and adiposity may obscure whether inflammation tracks the metabolic axis specifically or simply reflects excess adipose tissue; if hemogram-derived indices track metabolic burden independently of weight, they could help characterize metabolic–inflammatory status even in normal-weight women with PMOS, a group in whom metabolic risk is more easily overlooked.

This study therefore aimed to evaluate, in women with PMOS, the association between the TyG index and subclinical inflammation, reflected by CRP and the SII as primary markers (pre-specified in the ethics protocol) and by the NLR, PLR, MLR, and SIRI as secondary markers, testing whether these associations were independent of BMI. Secondary aims were to assess the correlation of the TyG index with HOMA-IR and fasting insulin and dose-response trends across TyG tertiles; as an exploratory aim, the discriminative performance of the TyG index for HOMA-IR-defined IR and the consistency of TyG-inflammation associations across BMI strata were evaluated. It was hypothesized that a higher TyG index would be associated with greater subclinical inflammation independently of adiposity, more consistently for hemogram-derived indices than for CRP. The hypothesis would have been refuted by the absence of an association between TyG and the hemogram-derived indices after adjustment for BMI or by an association of similar magnitude with CRP.

## 2. Materials and Methods

### 2.1. Study Design and Setting

This single-center, retrospective, cross-sectional study was conducted using clinical and laboratory data routinely collected as part of standard care from women with PMOS who attended a gynecology outpatient clinic between July 2025 and June 2026. The study was approved by the Clinical Research Ethics Committee of the study institution (approval no. 167-2026; approval date: 1 July 2026) and was conducted in accordance with the Declaration of Helsinki. The clinical data were thus generated during routine care before the study was approved; record screening and data extraction were performed, retrospectively, after ethics committee approval had been obtained. Individual informed consent was waived by the ethics committee given the retrospective, anonymized nature of the records. This study followed the STROBE guideline for cross-sectional studies [17]; the completed checklist is provided in Supplementary Table S1.

### 2.2. Participants

Following ethics committee approval, medical records of women with PMOS who attended the clinic during the study period were retrospectively screened (*n* = 189). All had been diagnosed according to the Rotterdam criteria, requiring at least two of oligo-anovulation, clinical hyperandrogenism, and polycystic ovarian morphology on ultrasonography [2]. In line with the 2023 international guideline, clinical hyperandrogenism was defined as hirsutism on clinical examination, acne, or female-pattern hair loss [2]; polycystic ovarian morphology was documented in all included women. Disease duration was defined as the time elapsed since the initial diagnosis. Inclusion criteria were age 18–45 years, a Rotterdam-criteria diagnosis, and complete biochemical/hematologic data. Exclusion criteria were overt diabetes mellitus, cardiovascular or chronic inflammatory/autoimmune disease, active infection, malignancy, pregnancy, use of statins or systemic glucocorticoids, and use of anti-inflammatory, insulin-sensitizing, or hormonal treatment within the preceding 3 months. Of the 189 women screened, 39 were excluded (12 diabetes mellitus, 15 hormonal treatment within the preceding 3 months, 2 active infection, and 10 incomplete data), leaving 150 women for analysis; no woman was using statins or systemic glucocorticoids. In the 10 women excluded for incomplete data, one or more laboratory tests had not been ordered or recorded at the index visit because of administrative or technical omissions (for example, a test not requested at the time of sampling), unrelated to the women’s clinical characteristics; these exclusions were made before any analysis.

### 2.3. Data Collection and Calculated Indices

Demographic, anthropometric, clinical, biochemical, hormonal, and hematologic data were extracted from patient records. As part of the routine diagnostic work-up, all blood samples (complete blood count, biochemistry, and hormonal profile) were drawn in the morning after an overnight fast on day 3 of a spontaneous menstrual cycle (early follicular phase); in women with no menstrual bleeding for more than 3 months (n = 24, 16.0%), samples were drawn on the day of presentation after the absence of a dominant follicle (≥10 mm in diameter) had been confirmed by transvaginal ultrasonography. Progestin-induced withdrawal bleeding was not used. Weight and height were measured at the same visit as blood sampling. BMI was calculated as weight (kg)/height (m)^2^. The TyG index was calculated as ln[fasting triglycerides (mg/dL) × fasting glucose (mg/dL)/2] [18]. Biochemical parameters, including fasting glucose, triglycerides, lipid fractions, glycated hemoglobin (HbA1c), and CRP, were measured on a Beckman Coulter AU2700 Plus clinical chemistry analyzer (Beckman Coulter Inc., Brea, CA, USA). CRP was measured by a latex particle-enhanced immunoturbidimetric assay (CRP Latex, OSR6199; Beckman Coulter) using the standard (non-high-sensitivity) application, with a reportable range of 0.5–480 mg/L and manufacturer-reported within-run and total coefficients of variation of 0.80–1.30% and 1.00–3.00%, respectively; values below the lower reportable limit were recorded as 0.5 mg/L. Fasting insulin and hormonal parameters (thyroid-stimulating hormone [TSH], follicle-stimulating hormone, luteinizing hormone, estradiol, prolactin, total and free testosterone, dehydroepiandrosterone sulfate, anti-Mullerian hormone [AMH], and sex hormone-binding globulin [SHBG]) were measured on a Beckman Coulter UniCel DxI 800 immunoassay analyzer, and complete blood counts were obtained on a Beckman Coulter DxH 900 hematology analyzer (both Beckman Coulter Inc., Brea, CA, USA). HOMA-IR was calculated as [fasting glucose (mg/dL) × fasting insulin (microIU/mL)]/405 [19]; insulin resistance was defined as HOMA-IR ≥ 2.5, the threshold used in previous Turkish and Chinese PMOS cohorts [9,11]. Hemogram-derived indices were calculated from complete blood count parameters: SII = platelet × neutrophil/lymphocyte count; NLR = neutrophil/lymphocyte; PLR = platelet/lymphocyte; MLR = monocyte/lymphocyte; and SIRI = neutrophil × monocyte/lymphocyte [16]. The SII and SIRI are expressed in units of 10^9^/L; the NLR, PLR, and MLR are dimensionless ratios.

### 2.4. Statistical Analysis

The sample size was determined a priori for the primary analysis, a multivariable linear regression of ln(inflammatory marker) on the TyG index, BMI, and age (one tested predictor and three total predictors). Using G*Power 3.1 (Heinrich-Heine-Universität Düsseldorf, Düsseldorf, Germany), with alpha = 0.05, 80% power, and assuming a small-to-moderate independent effect (f^2^ = 0.08, corresponding to a partial correlation of approximately 0.27), the required minimum sample size was 100 participants. This size was also adequate for the secondary bivariate correlations between the TyG index and CRP or the SII, for which 100 participants permit detection of correlations of rho ≥ 0.28 at alpha = 0.05 (two-sided) with 80% power. A minimum of 100 women was therefore planned, and all 150 eligible records available within the study period were included, exceeding the planned minimum. With 150 participants, the achieved power for the assumed effect was 93%, and correlations of rho ≥ 0.23 were detectable under the same assumptions. The observed adjusted effect for the SII (partial f^2^ = 0.67) was considerably larger than assumed, whereas the study was not powered to exclude small associations with CRP (partial f^2^ = 0.003).

Normality of continuous variables was assessed with the Shapiro–Wilk test; normally distributed variables are presented as the mean ± SD and non-normally distributed variables as the median (interquartile range, IQR), categorical variables as n (%). Bivariate associations between TyG and inflammatory/metabolic markers were assessed with Spearman correlation, with 95% confidence intervals (CIs) obtained by Fisher z-transformation; the correlation matrix is descriptive and was not corrected for multiple comparisons. The primary analysis, pre-specified in the ethics protocol, was multivariable linear regression of ln(CRP) and ln(SII) on the TyG index adjusted for age and BMI; identical models were fitted for the secondary markers. Markers were log-transformed because of right-skewed distributions. TyG coefficients are reported as unstandardized beta (per 1-unit increase in TyG) and as the percentage difference in the marker per 1-SD increment in TyG, calculated as [exp(beta × SD)—1] × 100, together with model R^2^. Model assumptions were examined by residual normality (Shapiro–Wilk), homoscedasticity (Breusch–Pagan test), linearity of the TyG term (added quadratic term), multicollinearity (variance inflation factor), and influential observations (Cook’s distance > 4/n); heteroscedasticity-consistent (HC3) standard errors and refitting after removal of influential observations were used as sensitivity checks.

To identify which component of the TyG index carried the associations, models were refitted with standardized ln(triglycerides) and fasting glucose entered together in place of TyG, and with HOMA-IR or fasting insulin in place of, and alongside, TyG. An extended sensitivity model additionally adjusted for free testosterone, SHBG, AMH, TSH, HbA1c, disease duration, and hirsutism. Because 19 CRP values were at the lower reportable limit, the CRP model was repeated with Tobit regression for left-censored data [20]. In a further sensitivity analysis, all regression models were repeated after excluding the 24 women sampled on the day of presentation. Participants were divided into TyG tertiles, with between-tertile differences assessed by the Kruskal–Wallis test and a Spearman-based trend test. Effect modification by adiposity was tested by adding a TyG × BMI interaction term (continuous BMI) to each model; in addition, TyG-marker correlations in normal-weight women (BMI < 25 kg/m^2^) and women with overweight/obesity (BMI ≥ 25 kg/m^2^) were compared with Fisher z tests. Women with overweight and obesity were combined because the overweight category was small (n = 16).

The correlations of TyG with HOMA-IR and fasting insulin were also examined. As an exploratory internal-consistency analysis, receiver operating characteristic (ROC) analysis (AUC, Youden index) evaluated the discriminative performance of TyG for HOMA-IR-defined insulin resistance. To determine whether the TyG index added information beyond routinely available variables, logistic regression models were compared: a baseline model containing BMI and age and the same model with the TyG index added. AUCs were compared with the DeLong test [21] and with 2000 bootstrap resamples stratified by insulin-resistance status.

Multiplicity was handled in two families. The two pre-specified primary markers (CRP and SII) were Bonferroni-corrected (significance threshold *p* < 0.025). The four secondary hemogram-derived indices (NLR, PLR, MLR, and SIRI) were corrected with the Benjamini–Hochberg procedure [22], with Bonferroni correction reported for comparison. The primary outcome and adjustment set were specified in the ethics protocol, whereas the composition of the correction families was defined at the analysis stage. Because the five hemogram-derived indices share numerators and denominators, the effective number of independent tests among them was estimated from the eigenvalues of their correlation matrix [23]. A two-sided *p* < 0.05 was considered significant unless stated otherwise. Analyses used Python (v3.13; Python Software Foundation, Wilmington, DE, USA; pandas 3.0, SciPy 1.18, statsmodels 0.15, and scikit-learn 1.9).

## 3. Results

### 3.1. Baseline Characteristics

The final cohort comprised 150 women with PMOS (Table 1). The median age was 28 years (IQR, 22–31), and the median BMI was 23.9 kg/m^2^ (IQR, 22.3–32.0; mean 26.9 ± 6.7 kg/m^2^), spanning normal-weight (59.3%), overweight (10.7%), and obese (30.0%) categories; the BMI distribution was right-skewed, with most women in the normal-weight range and a second cluster in the obese range (Supplementary Figure S1). The most frequent clinical features were menstrual irregularity (89.3%) and weight-related complaints (82.0%), followed by hair loss (75.3%), hirsutism (55.3%), acne (48.7%), and infertility (36.0%). Mean fasting glucose was 96.3 ± 9.1 mg/dL and mean triglycerides 143.6 ± 34.5 mg/dL, yielding a mean TyG index of 8.81 ± 0.32 (range, 7.98–9.49) (Table 2). The TyG index showed no significant correlation with BMI (rho = 0.03, 95% CI −0.13 to 0.19, *p* = 0.71). CRP values fell at the lower reportable limit of the assay (0.5 mg/L) in 19 out of 150 participants (12.7%).

### 3.2. Bivariate Correlations

**Figure 1:**
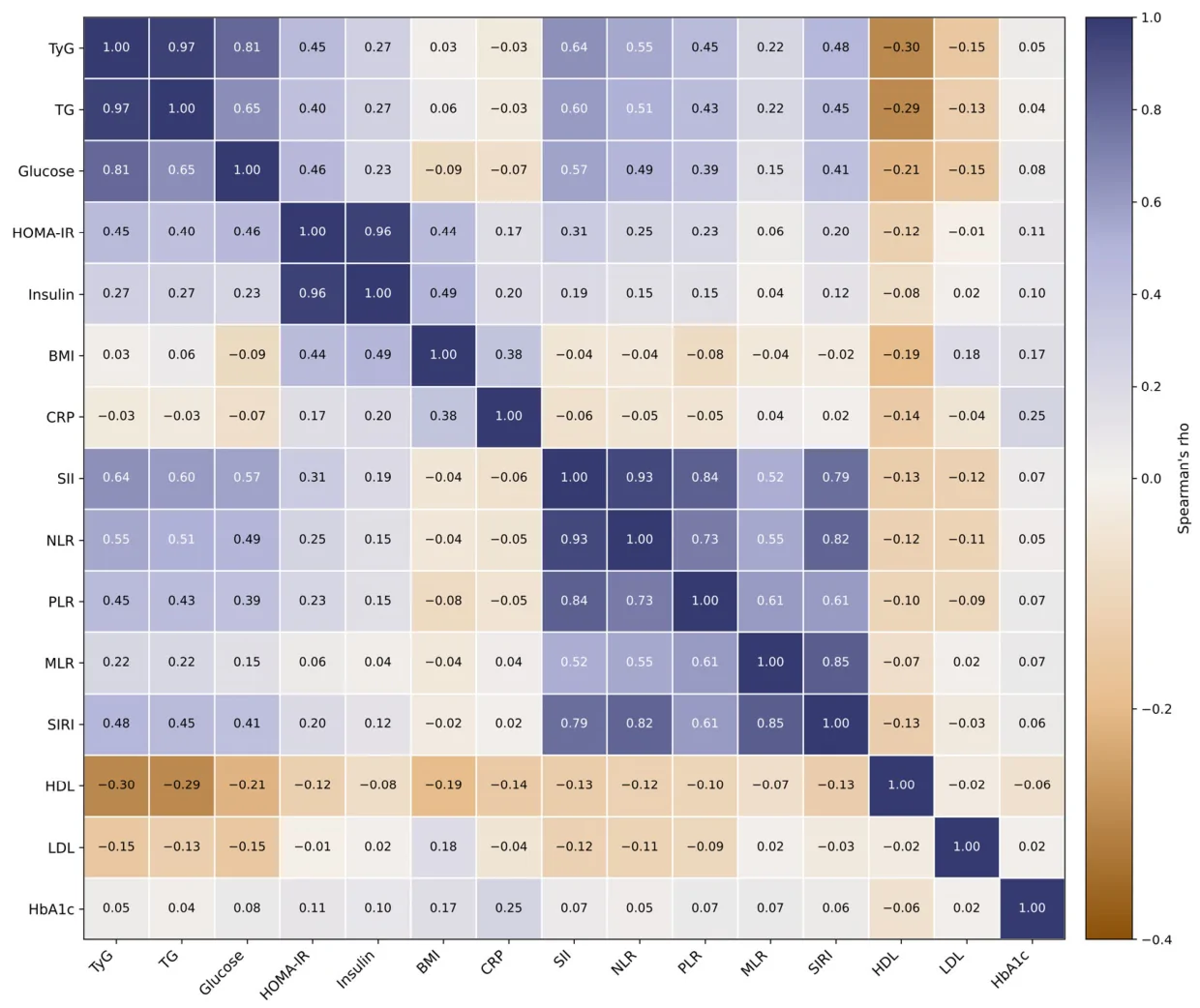
Spearman correlation matrix of the TyG index with metabolic, lipid, and inflammatory parameters. Cell values and color intensity represent Spearman’s rho; blue shading indicates a positive correlation and brown shading a negative correlation, with darker shading reflecting greater correlation strength; the color scale spans the observed range (−0.4 to 1.0). Coefficients are descriptive and were not corrected for multiple comparisons. TyG, triglyceride-glucose index; HOMA-IR, homeostatic model assessment of insulin resistance; BMI, body mass index; CRP, C-reactive protein; SII, systemic immune–inflammation index; NLR, neutrophil-to-lymphocyte ratio; PLR, platelet-to-lymphocyte ratio; MLR, monocyte-to-lymphocyte ratio; SIRI, systemic inflammation response index; HDL, high-density lipoprotein cholesterol; LDL, low-density lipoprotein cholesterol; HbA1c, glycated hemoglobin; Insulin, fasting insulin; TG, fasting triglycerides; Glucose, fasting glucose.

TyG correlated significantly with HOMA-IR (rho = 0.45, 95% CI 0.30 to 0.57, *p* < 0.001), a convergence partly inflated by their shared fasting glucose term; the correlation with fasting insulin alone was more modest (rho = 0.27, *p* < 0.001). BMI correlated in the expected directions with fasting insulin (rho = 0.49, *p* < 0.001), CRP (rho = 0.38, *p* < 0.001), and HDL cholesterol (rho = −0.19, *p* = 0.02) but not with fasting triglycerides (rho = 0.06, *p* = 0.44) or fasting glucose (rho = −0.09, *p* = 0.30), the two components of TyG (Figure 1). TyG correlated positively with the SII (rho = 0.64, 95% CI 0.53 to 0.74, *p* < 0.001), NLR (rho = 0.55, *p* < 0.001), SIRI (rho = 0.48, *p* < 0.001), PLR (rho = 0.45, *p* < 0.001), and MLR (rho = 0.22, *p* = 0.007) but not CRP (rho = −0.03, 95% CI −0.19 to 0.13, *p* = 0.70) (Table 3, Figure 1).

By contrast, HOMA-IR correlated more weakly with the hemogram-derived indices (rho = 0.31 for SII, 0.25 for NLR, 0.23 for PLR, 0.20 for SIRI, and 0.06 for MLR) and fasting insulin more weakly still (rho = 0.04 to 0.19) (Figure 1). Correlations among the composite hemogram indices were strong (SII-NLR rho = 0.93; SII-SIRI rho = 0.79), reflecting shared neutrophil and platelet components. TyG correlated inversely with high-density lipoprotein (HDL) cholesterol (rho = −0.30, *p* < 0.001), consistent with its triglyceride component, but showed no significant correlation with total cholesterol, low-density lipoprotein (LDL) cholesterol, or glycated hemoglobin (HbA1c) (all *p* > 0.05), suggesting that its inflammatory associations did not simply reflect global dyslipidemia or glycemic control (Figure 1).

### 3.3. Primary and Secondary Regression Analyses

After adjustment for age and BMI, TyG remained independently and positively associated with the primary marker SII (beta = 0.613, 95% CI 0.491 to 0.736, *p* < 0.001; Bonferroni-corrected *p* < 0.001), corresponding to a 21.7% (95% CI 17.0 to 26.6%) higher SII per 1-SD increment in TyG (model R^2^ = 0.41). TyG was not associated with the other primary marker, CRP (beta = −0.133, 95% CI −0.550 to 0.285, *p* = 0.53), whereas BMI was associated with CRP (beta = 0.058 per kg/m^2^, *p* < 0.001). Among the secondary markers, the corresponding per-SD differences were 13.9% for the NLR, 10.9% for the PLR, 15.1% for the SIRI, and 4.9% for the MLR (Table 3); the NLR, PLR, and SIRI remained significant under both Benjamini–Hochberg and Bonferroni correction (adjusted *p* < 0.001), whereas the MLR remained significant under Benjamini–Hochberg (adjusted *p* = 0.023) but not Bonferroni (adjusted *p* = 0.09), and its model explained little variance (R^2^ = 0.04). The effective number of independent tests among the five hemogram-derived indices was 3.0, and a Bonferroni threshold based on this number (*p* < 0.017) would likewise retain all indices except the MLR.

BMI showed small inverse associations with the SII (beta = −0.006 per kg/m^2^, 95% CI −0.012 to −0.0001, *p* = 0.047) and PLR (beta = −0.006, 95% CI −0.011 to −0.001, *p* = 0.015) but not with the NLR, MLR, or SIRI; in bivariate analyses BMI was uncorrelated with all hemogram-derived indices (|rho| ≤ 0.08) and with neutrophil, lymphocyte, and platelet counts (|rho| ≤ 0.07). Age was not associated with any marker (Table 3). Model diagnostics showed approximately normal and homoscedastic residuals for the hemogram-derived indices (Breusch–Pagan *p* ≥ 0.41), no departure from linearity (quadratic TyG term *p* ≥ 0.07), and no multicollinearity (variance inflation factors ≤ 1.01). Results were unchanged with HC3 robust standard errors, and the TyG coefficients increased slightly after removal of influential observations (e.g., SII beta = 0.657). In a Tobit model accounting for left-censoring of CRP, TyG remained unassociated with CRP (beta = −0.150, *p* = 0.53), whereas BMI remained associated (*p* < 0.001). After exclusion of the 24 women sampled on the day of presentation (n = 126), TyG remained associated with the SII (beta = 0.638, 95% CI 0.498 to 0.779, *p* < 0.001), NLR (beta = 0.444, *p* < 0.001), PLR (beta = 0.363, *p* < 0.001), MLR (beta = 0.158, *p* = 0.03), and SIRI (beta = 0.433, *p* < 0.001) and remained unassociated with CRP (beta = −0.054, *p* = 0.82).

**Figure 2:**
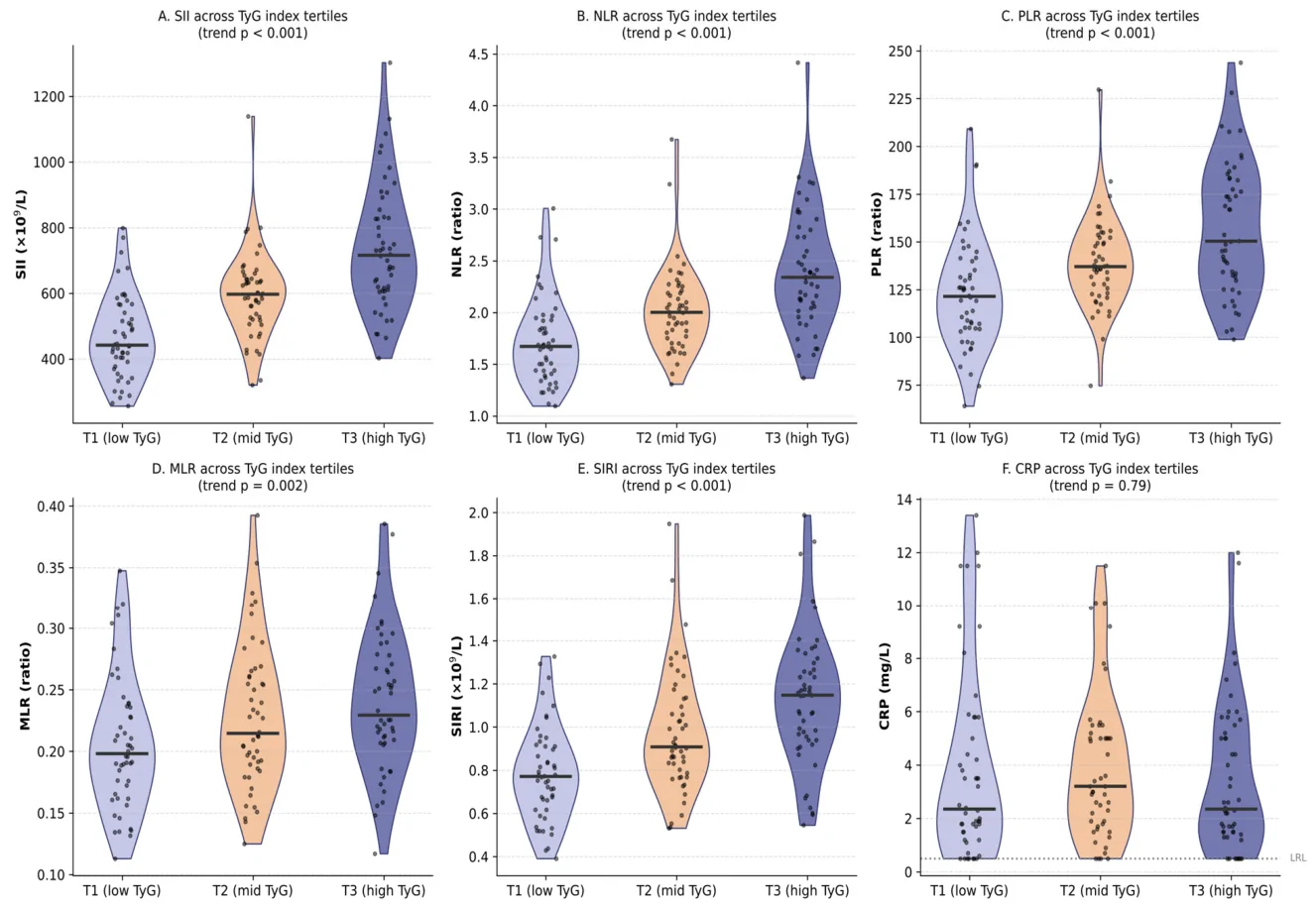
Distribution of systemic immune–inflammation index (SII), neutrophil-to-lymphocyte ratio (NLR), platelet-to-lymphocyte ratio (PLR), monocyte-to-lymphocyte ratio (MLR), and systemic inflammation response index (SIRI), and of C-reactive protein (CRP), across TyG index tertiles. Violin plots display the full distribution of each marker within TyG index tertiles (T1, lowest tertile; T2, middle tertile; T3, highest tertile); the horizontal line within each violin indicates the group median, and individual points represent single patients. The SII and SIRI are expressed in 10^9^/L, the NLR, PLR, and MLR are dimensionless ratios, and CRP is expressed in mg/L; the dotted line in panel F marks the lower reportable limit of the CRP assay (LRL, 0.5 mg/L). Trend *p* values are from the Spearman-based trend test. TyG, triglyceride-glucose index.

Across TyG tertiles, the SII, NLR, PLR, MLR, and SIRI increased stepwise with significant dose-response trends (all *p* < 0.01) (Figure 2; tertile medians and IQRs are given in Supplementary Table S2); CRP showed no significant difference or trend (Kruskal–Wallis *p* = 0.52; trend *p* = 0.79).

### 3.4. Contribution of the Triglyceride and Glucose Components and Extended Adjustment

When ln(triglycerides) and fasting glucose were entered together, both components were independently associated with the SII (per-SD differences of 12.8% and 10.2%, respectively; both *p* < 0.001) and NLR (7.7% and 7.3%; both *p* < 0.01), whereas the associations with the PLR, SIRI, and MLR were carried mainly by triglycerides (Table 4). HOMA-IR alone was associated with the SII, NLR, PLR, and SIRI but explained less variance than TyG (R^2^ = 0.16 vs. 0.41 for SII), and when TyG and HOMA-IR were entered together, TyG remained significant (*p* < 0.001 for SII), whereas HOMA-IR did not (all *p* ≥ 0.22) (Table 4). Fasting insulin alone showed weaker associations (per-SD differences of 2.5% to 9.8% for the hemogram-derived indices). After additional adjustment for free testosterone, SHBG, AMH, TSH, HbA1c, disease duration, and hirsutism, the TyG coefficients were materially unchanged (beta = 0.620 for SII, 0.411 for NLR, 0.318 for PLR, 0.147 for MLR, and 0.449 for SIRI; all *p* ≤ 0.03).

### 3.5. BMI-Stratified and Interaction Analyses

Stratifying the cohort into normal-weight (BMI < 25, n = 89) and overweight/obese (BMI ≥ 25, n = 61) subgroups (Table 5), the TyG-SII correlation was of comparable magnitude in both (rho = 0.67 vs. 0.62, both *p* < 0.001), as were correlations with the NLR (rho = 0.58 vs. 0.52, both *p* < 0.001) and SIRI (rho = 0.53 vs. 0.41, both *p* ≤ 0.001). These subgroup correlations are unadjusted, and the subgroups were not individually powered to detect between-stratum differences; formal tests, however, showed no evidence of effect modification, although the interaction for the PLR was borderline: the TyG × BMI interaction was not significant for any marker (*p* = 0.43 for SII, 0.84 for NLR, 0.06 for PLR, 0.66 for MLR, and 0.30 for SIRI), and correlations did not differ between strata (Fisher z *p* ≥ 0.35) (Table 5).

### 3.6. Exploratory Analysis of Discriminative Performance of the TyG Index for Insulin Resistance

**Figure 3:**
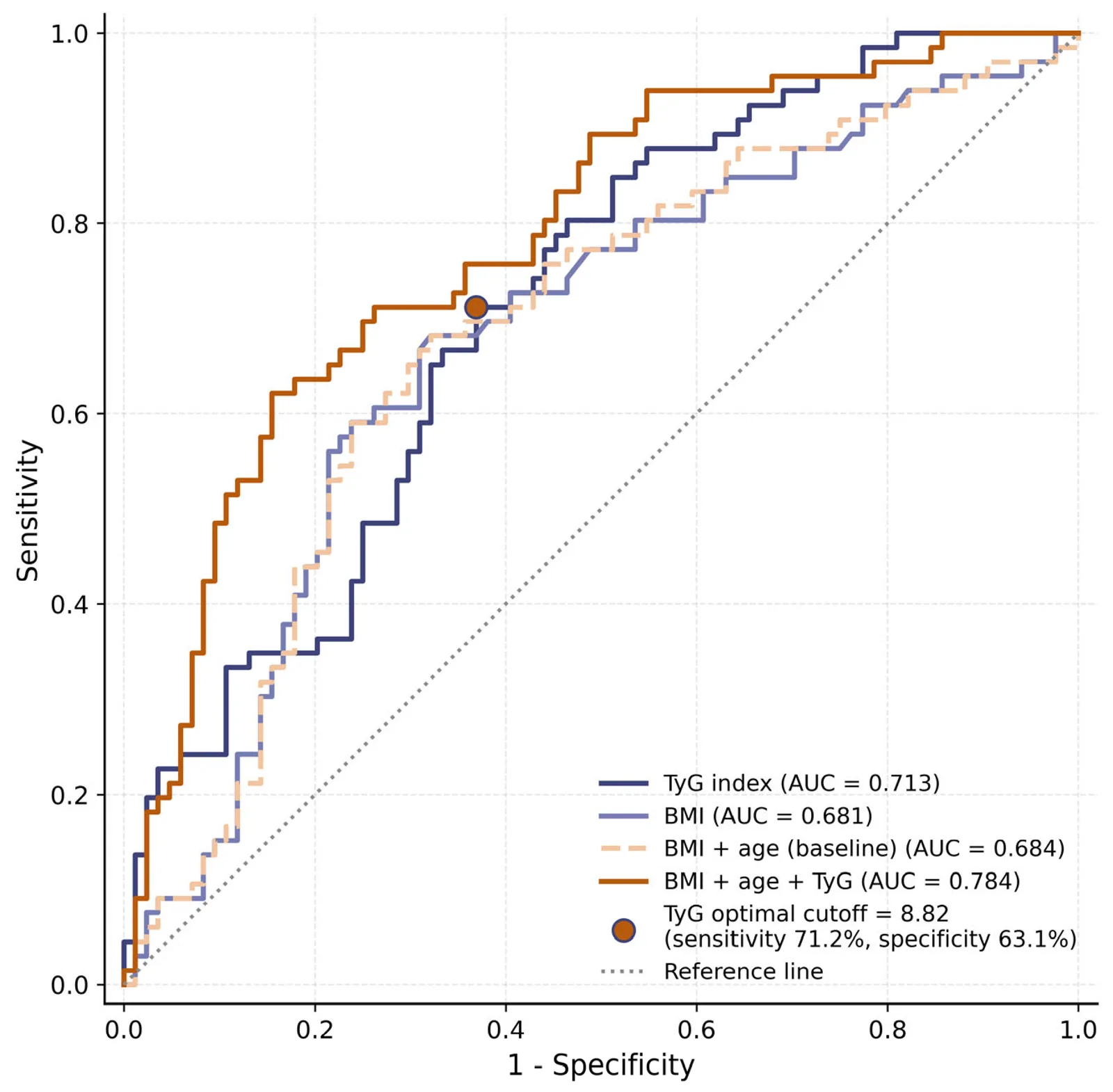
Receiver operating characteristic (ROC) curves for HOMA-IR-defined insulin resistance (HOMA-IR ≥ 2.5). Curves are shown for the TyG index alone, BMI alone, the baseline model containing BMI and age, and the baseline model with the TyG index added; the dotted diagonal line represents the line of no discrimination (reference line). The marked point indicates the optimal cutoff value for the TyG index by the Youden index. BMI, body mass index. TyG, triglyceride-glucose index; HOMA-IR, homeostatic model assessment of insulin resistance; AUC, area under the curve.

Because the TyG index and HOMA-IR share fasting glucose, this analysis is an internal-consistency analysis rather than validation against an independent criterion. ROC analysis evaluated the ability of TyG to identify HOMA-IR-defined insulin resistance (HOMA-IR ≥ 2.5), present in 44.0% of the cohort (n = 66) (Table 2). TyG showed moderate discrimination (AUC = 0.713; 95% CI, 0.63–0.79) (Figure 3), no better than that of fasting glucose alone (AUC = 0.737; DeLong *p* = 0.42) and higher than that of triglycerides alone (AUC = 0.684), indicating that much of the discrimination derives from the shared glucose term. The optimal Youden cutoff was 8.82, corresponding to 71.2% sensitivity and 63.1% specificity; because this value virtually coincides with the cohort mean (8.81), it divides the cohort roughly in half and should not be interpreted as a clinical threshold. Compared with routinely available variables, the TyG index alone did not discriminate better than anthropometry: the AUC was 0.681 for BMI alone and 0.684 for the baseline model containing BMI and age, and the difference between TyG alone and BMI alone was not significant (difference 0.032; 95% CI, −0.087 to 0.152; DeLong *p* = 0.60; stratified bootstrap *p* = 0.60); the comparable discrimination achieved by BMI alone is consistent with its correlation with fasting insulin (rho = 0.49), the insulin term of HOMA-IR. Adding the TyG index to the baseline model increased apparent discrimination from 0.684 to 0.784 (difference 0.10; 95% CI, 0.042 to 0.185; DeLong *p* = 0.011; stratified bootstrap *p* < 0.001); because this increment is also measured against a glucose-containing reference standard, it cannot be taken as evidence that TyG carries information on insulin resistance beyond age and adiposity (Figure 3).

## 4. Discussion

Among the 150 women with PMOS examined here, after adjustment for BMI and age, the TyG index remained independently associated with the hemogram-derived inflammatory indices SII, NLR, PLR, MLR, and SIRI but not with CRP. The association was strongest for the primary marker SII, was consistent across tertiles and BMI strata without evidence of effect modification, and persisted after additional adjustment for androgens, SHBG, AMH, TSH, HbA1c, and disease duration; the MLR showed the weakest association, which did not survive Bonferroni correction.

Decomposition of the index showed that both its triglyceride and glucose components contributed to these associations, whereas fasting insulin was only weakly related to the hemogram-derived indices, and HOMA-IR lost its association once TyG was in the model. The signal captured here is therefore better described as a triglyceride-glucose (metabolic) burden than as insulin resistance per se. Against routinely available variables, the TyG index alone did not outperform BMI in discriminating HOMA-IR-defined insulin resistance, and its apparent incremental value is partly structural because both measures contain fasting glucose.

These results suggest that the TyG index is more closely aligned with hemogram-derived inflammatory indices than with CRP, which in this cohort tracked adiposity instead. A plausible explanation lies in what each marker measures: the SII, NLR, PLR, MLR, and SIRI derive from circulating neutrophil, lymphocyte, platelet, and monocyte counts rather than a single hepatic acute-phase protein and therefore may be more sensitive to immune cell activation accompanying metabolic stress, whereas CRP synthesis is driven largely by adipose tissue mass [7]. This explanation is plausible but was not tested here, and the weak associations of HOMA-IR and fasting insulin with the hemogram-derived indices argue against insulin resistance as the main link. Although large population analyses have linked the SII to insulin resistance [24], in the present cohort, the hemogram-derived indices were more closely related to TyG than to HOMA-IR or fasting insulin.

The TyG-HOMA-IR correlation observed (rho = 0.45) falls within the range reported in other PMOS cohorts (0.37–0.52) [9,10,11], although those studies used Pearson [9,11] or Spearman [10] coefficients, which are not directly comparable with the present estimate; the moderate correlation, which is partly inflated by the shared glucose term, indicates that the two indices are not interchangeable [25]. The AUC of the exploratory ROC analysis (0.713) is not directly comparable with the value of 0.781 reported in a Chinese PMOS cohort [11] because in both analyses, the reference standard shares the glucose term with TyG.

Earlier case-control work reported a higher SII in women with PMOS than in controls [14]; the present within-cohort design cannot address such between-group differences but shows that, among women with PMOS, hemogram-derived indices vary with the triglyceride-glucose axis regardless of weight status. This is consistent with meta-analytic data showing higher TyG values in PMOS [26] and with evidence linking the SII to dyslipidemia in this population [27].

Mechanistically, higher triglyceride and fasting glucose levels, the two components of TyG, may promote a mild pro-inflammatory, pro-oxidant state reflected in leukocyte and platelet indices rather than in a single acute-phase reactant such as CRP [12], consistent with a study of adults with obesity and metabolic syndrome linking elevated triglyceride and CRP to greater oxidative stress and reduced antioxidant capacity [28]. Neutrophilia, relative lymphopenia, and mild thrombocytosis, the components underlying the SII, NLR, PLR, and SIRI, have each been linked to metabolic dysfunction outside PMOS, including a meta-analysis of over 70,000 individuals confirming a higher NLR in metabolic syndrome [29]. Apart from the expected inverse association with HDL cholesterol, TyG was unrelated to total cholesterol, LDL cholesterol, and HbA1c, suggesting that this relationship reflects the triglyceride-glucose interplay specifically rather than generalized dyslipidemia or glycemic burden. This does not imply that CRP is uninformative in PMOS: a meta-analysis found CRP elevated in PMOS even among women without obesity [6], whereas in this cohort, CRP variation was explained predominantly by BMI. An elevated NLR has also been reported in PMOS [30], and the SIRI has been associated with metabolic disorders in the general population [31].

Clinically, the low cost and routine availability of TyG and hemogram-derived indices make them attractive candidates for further study as markers of metabolic–inflammatory status in PMOS, with the qualification that their value appears to lie in supplementing rather than replacing anthropometric assessment. Longitudinal data link TyG to future atherosclerotic cardiovascular disease [32] and cardiovascular risk alongside related lipid ratios [33]; separately, the SII and SIRI have been tied to long-term cardiovascular mortality [34]; whether these associations apply to women with PMOS, and whether TyG-associated inflammation carries prognostic value, remains to be established.

The absence of an association between the TyG index and BMI in this cohort is notable, given that both are linked to adiposity-related metabolic pathways. It is unlikely to reflect a measurement artifact: the TyG index showed the expected inverse relationship with HDL cholesterol (rho = −0.30) and a positive relationship with HOMA-IR (rho = 0.45), while BMI itself correlated in the expected directions with fasting insulin, CRP, and HDL cholesterol. A more plausible explanation lies in cohort composition: 59.3% of participants were of normal weight, a group in which insulin resistance frequently occurs independently of body mass [35], so that variation in TyG within this cohort was driven more by metabolic than by anthropometric differences. The right-skewed BMI distribution, with few women with overweight (Supplementary Figure S1), may also have attenuated the correlation.

Relatedly, BMI showed small inverse associations with the SII and PLR in the adjusted models. Because TyG and BMI were uncorrelated, these are unlikely to reflect statistical suppression, and because BMI was uncorrelated with the individual cell counts, we regard them as a cohort-specific finding of uncertain significance rather than as evidence of an inverse adiposity-inflammation relationship.

This study has several strengths. Blood samples were obtained under standardized conditions (morning, fasting, and, in women with spontaneous menses, early follicular phase) and analyzed in a single laboratory; the primary outcome and adjustment set were specified in the ethics protocol; and the main findings were robust to extended adjustment for hormonal and metabolic covariates, robust standard errors, exclusion of influential observations, censored-data modeling of CRP, and exclusion of women sampled on the day of presentation. Because all hemogram-derived indices come from a routine complete blood count, the results are readily applicable in clinical settings.

Several limitations warrant mention. The cross-sectional, retrospective, single-center design precludes causal inference and limits generalizability, and the absence of a non-PMOS control group confines all associations to variation within the PMOS cohort. Physical activity, diet, alcohol intake, smoking status, and blood pressure were not available in the clinical records; because lifestyle factors influence both triglyceride levels and leukocyte counts, residual confounding cannot be excluded. Hirsutism was assessed clinically without a modified Ferriman–Gallwey score, and the HOMA-IR threshold of 2.5, although used in previous PMOS cohorts [9,11], has not been validated against clamp measurements in Turkish women. The inverse BMI coefficients for SII and PLR remain unexplained, and the composition of the multiplicity-correction families was defined at the analysis stage.

CRP was measured with a standard rather than a high-sensitivity assay, and 12.7% of values were at the lower reportable limit; although a Tobit model accounting for censoring gave the same result, correlations weaker than approximately 0.20 could not be excluded (95% CI −0.19 to 0.13), and the study was not powered to detect small associations with CRP. The null TyG-CRP association should therefore be read as an absence of detectable association under these measurement conditions and the contrast with the hemogram-derived indices as hypothesis-generating. The hemogram-derived indices were strongly intercorrelated (approximately three effective independent tests), and MLR, which survived only Benjamini–Hochberg correction, should be interpreted cautiously. Finally, because TyG and HOMA-IR share fasting glucose, their convergence and the discriminative accuracy of TyG against a HOMA-IR-defined reference standard are partly structural and do not constitute validation against an independent criterion.

Taken together, these data suggest that the TyG index is a practical marker of a triglyceride-glucose (metabolic)–inflammatory axis in PMOS that, in this cohort, was largely independent of adiposity and was reflected in hemogram-derived inflammatory indices. They also caution against treating CRP alone as sufficient to capture this relationship. Prospective, multicenter studies incorporating high-sensitivity CRP, direct measures of insulin sensitivity, and cytokine panels are warranted to clarify the temporal and mechanistic relationship between metabolic burden and systemic inflammation in PMOS and to evaluate whether TyG-guided risk stratification could inform earlier metabolic screening, particularly among normal-weight women in whom metabolic risk is more easily overlooked.

## 5. Conclusions

In this cohort of women with PMOS, the TyG index was associated with hemogram-derived inflammatory indices, most strongly with the SII, independently of BMI, with consistent findings across BMI strata and after extended adjustment, whereas CRP was associated with adiposity rather than with TyG. The association was attributable to the triglyceride and glucose components of the index rather than to fasting insulin. These findings support further evaluation of TyG-based indices, derived from routine and inexpensive laboratory tests, as accessible tools for capturing the interplay between metabolic burden and subclinical inflammation in PMOS and suggest that CRP alone may be insufficient to represent this relationship. Confirmation in prospective, controlled studies is needed before these indices can be recommended for routine risk stratification.

## Figures and Tables

**Table 1:** Baseline demographic and clinical characteristics of the study population.

Characteristic	Value (n = 150)
Age (years), median (IQR)	28 (22–31)
BMI (kg/m^2^), median (IQR)	23.9 (22.3–32.0)
BMI (kg/m^2^), mean ± SD	26.9 ± 6.7
Normal weight (<25 kg/m^2^), n (%)	89 (59.3)
Overweight (25–29.9 kg/m^2^), n (%)	16 (10.7)
Obese (≥30 kg/m^2^), n (%)	45 (30.0)
Time since diagnosis, 1–5 years, n (%)	122 (81.3)
Time since diagnosis, 5–10 years, n (%)	28 (18.7)
Irregular menses, n (%)	134 (89.3)
Weight-related complaints, n (%)	123 (82.0)
Hair loss, n (%)	113 (75.3)
Hirsutism, n (%)	83 (55.3)
Acne, n (%)	73 (48.7)
Infertility, n (%)	54 (36.0)

BMI, body mass index; PMOS, polyendocrine metabolic ovarian syndrome; IQR, interquartile range; SD, standard deviation. Age and BMI were non-normally distributed (Shapiro–Wilk *p* < 0.01).

**Table 2:** Laboratory and biochemical characteristics of the study population.

Parameter	Value (n = 150)
Fasting glucose (mg/dL), mean ± SD	96.3 ± 9.1
Fasting triglycerides (mg/dL), mean ± SD	143.6 ± 34.5
TyG index, mean ± SD	8.81 ± 0.32
Fasting insulin (microIU/mL), mean ± SD	9.66 ± 3.24
HOMA-IR, mean ± SD	2.31 ± 0.84
Insulin-resistant (HOMA-IR ≥ 2.5), n (%)	66 (44.0)
CRP (mg/L), median (IQR)	2.65 (1.50–5.20)
SII, median (IQR)	586.1 (470.5–681.2)
NLR, median (IQR)	1.97 (1.67–2.31)
PLR, median (IQR)	135.5 (118.9–155.6)
MLR, median (IQR)	0.22 (0.19–0.26)
SIRI, median (IQR)	0.92 (0.75–1.16)

TyG, triglyceride-glucose index; HOMA-IR, homeostatic model assessment of insulin resistance; CRP, C-reactive protein; SII, systemic immune–inflammation index; NLR, neutrophil-to-lymphocyte ratio; PLR, platelet-to-lymphocyte ratio; MLR, monocyte-to-lymphocyte ratio; SIRI, systemic inflammation response index; IQR, interquartile range; SD, standard deviation. Variables with a normal distribution (Shapiro–Wilk *p* > 0.05) are presented as the mean ± SD and those with a non-normal distribution as the median (IQR); fasting insulin (Shapiro–Wilk *p* = 0.15) and HOMA-IR (*p* = 0.19) met the normality criterion. CRP was measured with a standard (non-high-sensitivity) assay; 19 values (12.7%) were at the lower reportable limit of 0.5 mg/L. The SII and SIRI are expressed in 10^9^/L; the NLR, PLR, and MLR are dimensionless.

**Table 3:** Unadjusted correlations, tertile trends, and full multivariable models of the TyG index, BMI, and age for each inflammatory marker.

Marker	Unadjusted Spearman Rho (*p*)	Tertile Trend *p*	TyG index, Beta (95% CI); *p*; % per SD	BMI, Beta (95% CI); *p*	Age, Beta (95% CI); *p*
CRP (R^2^ = 0.20)	−0.03 (0.70)	0.79	−0.133 (−0.550 to 0.285); 0.53; −4.2%	0.058 (0.038 to 0.078); <0.001	−0.016 (−0.040 to 0.007); 0.17
SII (R^2^ = 0.41)	0.64 (<0.001)	<0.001	0.613 (0.491 to 0.736); <0.001; +21.7%	−0.006 (−0.012 to −0.0001); 0.047	−0.000 (−0.007 to 0.006); 0.90
NLR (R^2^ = 0.27)	0.55 (<0.001)	<0.001	0.405 (0.294 to 0.516); <0.001; +13.9%	−0.004 (−0.010 to 0.001); 0.13	0.000 (−0.006 to 0.006); 0.95
PLR (R^2^ = 0.24)	0.45 (<0.001)	<0.001	0.324 (0.221 to 0.428); <0.001; +10.9%	−0.006 (−0.011 to −0.001); 0.015	−0.003 (−0.009 to 0.003); 0.28
MLR (R^2^ = 0.04)	0.22 (0.007)	0.002	0.150 (0.021 to 0.279); 0.023; +4.9%	−0.003 (−0.010 to 0.003); 0.27	0.001 (−0.006 to 0.009); 0.71
SIRI (R^2^ = 0.20)	0.48 (<0.001)	<0.001	0.439 (0.293 to 0.586); <0.001; +15.1%	−0.003 (−0.010 to 0.004); 0.37	0.004 (−0.004 to 0.012); 0.32

Each row presents a separate multivariable linear regression of ln(marker) on the TyG index, BMI, and age (n = 150); unadjusted correlations are Spearman coefficients, and tertile trend *p* values are from a Spearman-based trend test across TyG index tertiles. CRP, C-reactive protein; SII, systemic immune–inflammation index; NLR, neutrophil-to-lymphocyte ratio; PLR, platelet-to-lymphocyte ratio; MLR, monocyte-to-lymphocyte ratio; SIRI, systemic inflammation response index; CI, confidence interval; R^2^, coefficient of determination; SD, standard deviation. Percentages give the difference in the marker per 1-SD (0.32-unit) higher TyG. CRP and the SII were the pre-specified primary markers (Bonferroni-adjusted across the two: *p* < 0.001 for the SII and *p* = 1.00 for CRP). For the secondary markers, Benjamini–Hochberg-adjusted *p* < 0.001 for the NLR, PLR, and SIRI and *p* = 0.023 for the MLR; after Bonferroni correction, the MLR no longer reached significance (adjusted *p* = 0.09), whereas the NLR, PLR, and SIRI remained significant.

**Table 4:** Associations of the triglyceride and glucose components of the TyG index and of insulin-based measures with inflammatory markers.

Marker	ln(TG), % per SD (95% CI); *p*	Fasting Glucose, % per SD (95% CI); *p*	HOMA-IR Alone, % per SD; *p*	Fasting Insulin Alone, % per SD; *p*	HOMA-IR with TyG in Model, % per SD; *p*
CRP	−4.7 (−20.6 to +14.3); 0.60	+0.4 (−16.2 to +20.4); 0.96	−0.9; 0.91	+1.0; 0.90	+2.1; 0.81
SII	+12.8 (+6.9 to +18.9); <0.001	+10.2 (+4.5 to +16.2); <0.001	+14.2; <0.001	+9.8; 0.001	+3.1; 0.23
NLR	+7.7 (+2.7 to +13.0); 0.003	+7.3 (+2.2 to +12.5); 0.005	+8.6; <0.001	+5.6; 0.02	+1.3; 0.59
PLR	+7.5 (+2.7 to +12.4); 0.002	+4.2 (−0.4 to +9.0); 0.07	+8.1; <0.001	+6.0; 0.006	+2.7; 0.22
MLR	+5.8 (+0.0 to +11.9); 0.05	−0.8 (−6.2 to +4.9); 0.79	+3.0; 0.21	+2.5; 0.30	+0.4; 0.89
SIRI	+11.0 (+4.1 to +18.3); 0.002	+4.9 (−1.5 to +11.8); 0.14	+8.9; 0.003	+6.2; 0.04	+0.8; 0.80

Values are percentage differences in the marker per 1-SD higher predictor from linear regression of ln(marker) adjusted for age and BMI (n = 150). ln(TG) and fasting glucose were entered together in one model; HOMA-IR and fasting insulin were each entered alone in place of TyG; the last column gives HOMA-IR estimates from a model that also contained TyG. TG, triglycerides; HOMA-IR, homeostatic model assessment of insulin resistance; SD, standard deviation; CI, confidence interval; other abbreviations as in Table 3.

**Table 5:** BMI-stratified Spearman correlations between the TyG index and inflammatory markers, with tests for effect modification.

Marker	Normal Weight (*n* = 89), Rho (95% CI); *p*	Overweight/Obese (*n* = 61), Rho (95% CI); *p*	*p* for Difference (Fisher z)	*p* for TyG × BMI Interaction
CRP	−0.05 (−0.26 to 0.16); 0.64	−0.11 (−0.36 to 0.14); 0.38	0.71	0.53
SII	0.67 (0.52 to 0.78); <0.001	0.62 (0.41 to 0.76); <0.001	0.63	0.43
NLR	0.58 (0.41 to 0.71); <0.001	0.52 (0.29 to 0.69); <0.001	0.62	0.84
PLR	0.40 (0.20 to 0.57); <0.001	0.52 (0.29 to 0.69); <0.001	0.41	0.06
MLR	0.21 (0.00 to 0.40); 0.05	0.22 (−0.04 to 0.45); 0.09	0.95	0.66
SIRI	0.53 (0.35 to 0.68); <0.001	0.41 (0.16 to 0.60); 0.001	0.35	0.30

SII, systemic immune–inflammation index; NLR, neutrophil-to-lymphocyte ratio; SIRI, systemic inflammation response index; BMI, body mass index; CRP, C-reactive protein; PLR, platelet-to-lymphocyte ratio; MLR, monocyte-to-lymphocyte ratio; CI, confidence interval. Interaction *p* values are from linear regression of ln(marker) on TyG, BMI (continuous), their product term, and age.

## Data Availability

The data presented in this study are available in Zenodo at https://doi.org/10.5281/zenodo.23110622 under restricted access. These data are available from the corresponding author upon reasonable request due to privacy restrictions.

## Supplementary Materials

The following supporting information can be downloaded at https://www.mdpi.com/article/10.3390/metabo16100771/s1: Table S1: STROBE checklist; Table S2: Inflammatory markers across TyG index tertiles; Figure S1: Distribution of body mass index.
