# Supplementary material for: Triglyceride-Glucose Index and Systemic Inflammatory Markers in Polyendocrine Metabolic Ovarian Syndrome (Polycystic Ovary Syndrome): A Cross-Sectional Study
Source: Metabolites. 2026 Oct 12;16(10):771. doi: 10.3390/metabo16100771 (PMC13706786; doi:10.3390/metabo16100771)
Supplement: Supplementary file 1 [file metabolites-16-00771-s001.zip › metabolites-4598889-supplementary.pdf]

Supplementary Materials

Triglyceride-Glucose Index and Systemic Inflammatory Markers in Polyendocrine Metabolic Ovarian Syndrome (Polycystic Ovary Syndrome): A Cross-Sectional Study

Meryem Kececi Oguzhanoglu, Kursat Oguzhanoglu

Table S1. STROBE statement: checklist of items that should be included in reports of cross-sectional studies.

Section/Topic	Item	Recommendation	Reported in manuscript (section, lines)
Title and abstract	1	(a) Indicate the study's design with a commonly used term in the title or the abstract	Title (lines 1–3); Abstract (line 37)
		(b) Provide in the abstract an informative and balanced summary of what was done and what was found	Abstract (lines 32–55)
**Introduction**			
Background/rationale	2	Explain the scientific background and rationale for the investigation being reported	Introduction (lines 59–95)
Objectives	3	State specific objectives, including any prespecified hypotheses	Introduction (lines 96–107)
**Methods**			
Study design	4	Present key elements of study design early in the paper	Section 2.1 (lines 110–114)
Setting	5	Describe the setting, locations, and relevant dates, including periods of recruitment, exposure, follow-up, and data collection	Section 2.1 (lines 110–120)
Participants	6	(a) Give the eligibility criteria, and the sources and methods of selection of participants	Section 2.2 (lines 122–135)
Variables	7	Clearly define all outcomes, exposures, predictors, potential confounders, and effect modifiers. Give diagnostic criteria, if applicable	Sections 2.2–2.4 (lines 122–168; lines 183–211)
Data sources/measurement	8*	For each variable of interest, give sources of data and details of methods of assessment (measurement)	Section 2.3 (lines 141–168)
Bias	9	Describe any efforts to address potential sources of bias	Section 2.3 (lines 142–148); Section 2.4 (lines 194–211); Discussion (lines 496–505)
Study size	10	Explain how the study size was arrived at	Section 2.4 (lines 170–182)
Quantitative variables	11	Explain how quantitative variables were handled in the analyses. If applicable, describe which groupings were chosen and why	Section 2.4 (lines 183–211)
Statistical methods	12	(a) Describe all statistical methods, including those used to control for confounding	Section 2.4 (lines 183–228)
		(b) Describe any methods used to examine subgroups and interactions	Section 2.4 (lines 207–211)
		(c) Explain how missing data were addressed	Section 2.2 (lines 129–139); complete-case analysis
		(d) If applicable, describe analytical methods taking account of sampling strategy	Not applicable (consecutive single-center records)
		(e) Describe any sensitivity analyses	Section 2.4 (lines 194–211)
**Results**			

Section/Topic	Item	Recommendation	Reported in manuscript (section, lines)
Participants	13*	(a) Report numbers of individuals at each stage of study	Section 2.2 (lines 133–135): 189 screened, 39 excluded, 150 analyzed
		(b) Give reasons for non-participation at each stage	Section 2.2 (lines 133–135)
		(c) Consider use of a flow diagram	Not provided; numbers and reasons are given in the text
Descriptive data	14*	(a) Give characteristics of study participants and information on exposures and potential confounders	Section 3.1 (lines 231–241); Tables 1 and 2
		(b) Indicate number of participants with missing data for each variable of interest	None in the analyzed cohort (complete data was an inclusion criterion; lines 129–130)
Outcome data	15*	Report numbers of outcome events or summary measures	Section 3.1; Table 2
Main results	16	(a) Give unadjusted estimates and, if applicable, confounder-adjusted estimates and their precision (e.g., 95% CI). Make clear which confounders were adjusted for and why they were included	Sections 3.2–3.3 (lines 257–316); Table 3
		(b) Report category boundaries when continuous variables were categorized	Table 1 (BMI categories); Supplementary Table S2 (TyG tertile ranges)
		(c) If relevant, consider translating estimates of relative risk into absolute risk for a meaningful time period	Not applicable
Other analyses	17	Report other analyses done, e.g., analyses of subgroups and interactions, and sensitivity analyses	Sections 3.3–3.6 (lines 302–403); Tables 4 and 5
**Discussion**			
Key results	18	Summarise key results with reference to study objectives	Discussion (lines 414–426)
Limitations	19	Discuss limitations of the study, taking into account sources of potential bias or imprecision. Discuss both direction and magnitude of any potential bias	Discussion (lines 496–516)
Interpretation	20	Give a cautious overall interpretation of results considering objectives, limitations, multiplicity of analyses, results from similar studies, and other relevant evidence	Discussion (lines 427–487; lines 517–525)
Generalisability	21	Discuss the generalisability (external validity) of the study results	Discussion (lines 496–498)
**Other information**			
Funding	22	Give the source of funding and the role of the funders for the present study and, if applicable, for the original study on which the present article is based	Funding statement (line 546)

**Give information separately for exposed and unexposed groups, if applicable. Line numbers refer to the revised manuscript.*

Table S2. Inflammatory markers across TyG index tertiles.

Marker	T1 (n = 50)<br>TyG 7.981–8.705	T2 (n = 50)<br>TyG 8.721–8.958	T3 (n = 50)<br>TyG 8.959–9.494	Kruskal-Wallis p	Trend p
CRP (mg/L)	2.35 (1.50–5.60)	3.20 (1.82–5.15)	2.35 (1.50–5.15)	0.52	0.79
SII (×10^9/L)	441.8 (380.5–558.6)	597.0 (521.6–643.9)	715.9 (608.3–831.6)	<0.001	<0.001
NLR	1.67 (1.41–1.93)	2.00 (1.76–2.21)	2.34 (2.05–2.73)	<0.001	<0.001
PLR	121.4 (104.8–138.7)	137.0 (123.8–152.3)	150.3 (131.9–182.9)	<0.001	<0.001
MLR	0.20 (0.17–0.24)	0.21 (0.19–0.26)	0.23 (0.21–0.28)	0.007	0.002
SIRI (×10^9/L)	0.77 (0.63–0.91)	0.91 (0.79–1.12)	1.15 (0.94–1.29)	<0.001	<0.001

Values are median (interquartile range). TyG index tertiles were defined by the 33.3rd and 66.7th percentiles of the TyG distribution (8.715 and 8.958); tertile ranges are given to three decimal places in the column headings, and no observation fell on a boundary value, so the tertiles do not overlap. Trend p values are from a Spearman-based trend test across tertiles. CRP was measured with a standard (non-high-sensitivity) assay; values below the lower reportable limit were recorded as 0.5 mg/L. NLR, PLR, and MLR are dimensionless ratios. TyG, triglyceride-glucose index; CRP, C-reactive protein; SII, systemic immune-inflammation index; NLR, neutrophil-to-lymphocyte ratio; PLR, platelet-to-lymphocyte ratio; MLR, monocyte-to-lymphocyte ratio; SIRI, systemic inflammation response index.

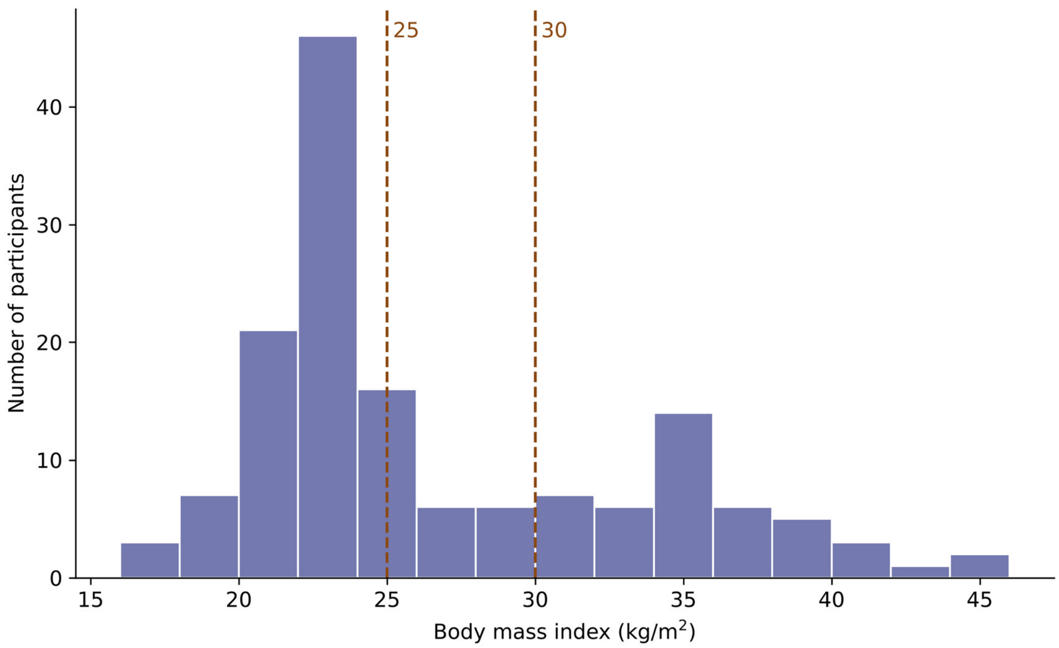

Figure S1. Distribution of body mass index (BMI) in the study cohort (n = 150). Dashed lines mark the 25 and 30 kg/m² thresholds for overweight and obesity. The distribution is right-skewed, with most women in the normal-weight range, a second cluster in the obese range, and few women in the overweight range (n = 16).
